# Comparison of methacholine and mannitol challenges: importance of method of methacholine inhalation

**DOI:** 10.1186/s13223-020-0410-x

**Published:** 2020-02-11

**Authors:** Donald W. Cockcroft, Beth E. Davis, Christianne M. Blais

**Affiliations:** 1 Department of Medicine, University of Saskatchewan, Saskatoon, SK Canada; 2 Royal University Hospital, 103 Hospital Drive, Saskatoon, SK S7N0W8 Canada

**Keywords:** Methacholine inhalation test, Deep inhalation (TLC) method, Tidal breathing method, Mannitol inhalation test, Sensitivity, Specificity

## Abstract

**Background:**

Direct inhalation challenges (e.g. methacholine) are stated to be more sensitive and less specific for a diagnosis of asthma than are indirect challenges (e.g. exercise, non-isotonic aerosols, mannitol, etc.). However, data surrounding comparative sensitivity and specificity for methacholine compared to mannitol challenges are conflicting. When methacholine is inhaled by deep total lung capacity (TLC) inhalations, deep inhalation inhibition of bronchoconstriction leads to a marked loss of diagnostic sensitivity when compared to tidal breathing (TB) inhalation methods. We hypothesized that deep inhalation methacholine methods with resulting bronchoprotection may be the explanation for conflicting sensitivity/specificity data.

**Methods:**

We reviewed 27 studies in which methacholine and mannitol challenges were performed in largely the same individuals. Methacholine was inhaled by dosimeter TLC methods in 13 studies and by tidal breathing in 14 studies. We compared the rates of positive methacholine (stratified by inhalation method) and mannitol challenges in both asthmatics and non-asthmatics.

**Results:**

When methacholine was inhaled by TLC inhalations the prevalence of positive tests in asthmatics, 60.2% (548/910), was similar to mannitol, 58.9% (537/912). By contrast, when methacholine was inhaled by tidal breathing the prevalence of positive tests in asthmatics 83.1% (343/413) was more than double that of mannitol, 41.5% (146/351). In non-asthmatics, the two methacholine methods resulted in positive tests in 18.8% (142/756) and 16.2% (27/166) by TLC and TB inhalations respectively. This compares to an overall 8.3% (n = 76) positive rate for mannitol in 913 non-asthmatics.

**Conclusion:**

These data support the hypothesis that the conflicting data comparing methacholine and mannitol sensitivity and specificity are due to the method of methacholine inhalation. Tidal breathing methacholine methods have a substantially greater sensitivity for a diagnosis of asthma than either TLC dosimeter methacholine challenge methods or mannitol challenge. Methacholine challenges should be performed by tidal breathing as per recent guideline recommendations. Methacholine (more sensitive) and mannitol (more specific) will thus have complementary diagnostic features.

## Background

Measurement of non-allergic or non-specific airway hyperresponsiveness (AHR) is a valuable tool in the clinical assessment of patients with possible asthma, those with asthma-like symptoms and non-diagnostic, generally normal, lung function. Stimuli used to measure AHR have been classified as direct and indirect [1]. Direct stimuli act directly on airway smooth muscle receptors; examples include methacholine acting on muscarinic receptors and histamine acting on H_1_ receptors. Indirect stimuli act through one or more intermediate pathways most via mediators released from metachromatic inflammatory cells (mast cells, basophils); examples include exercise, eucapnic voluntary hyperpnea (EVH), non-isotonic aerosols, propranolol, adenosine monophosphate (AMP) and dry powder mannitol [2]. Direct AHR reflects airway smooth muscle function, perhaps modulated by inflammation, while indirect AHR reflects airway inflammation [1, 2]. The consensus is that direct AHR is highly sensitive for current asthma whereas indirect AHR is highly specific while being relatively insensitive particularly for mild and/or well controlled asthma [2].

Dry powder mannitol (Aridol^®^) inhalation is an indirect challenge test [3] with several advantages. The advantages include the dose–response nature of the test (in contrast particularly to exercise and EVH), the lack of requirement for expensive and bulky equipment, and the fact that there is only a single method for administration of mannitol. In addition, we suspect that the mannitol challenge is less likely to be dose limited compared to other indirect challenges such as exercise, EVH, propranolol or AMP.

Studies comparing the diagnostic properties of the direct methacholine challenge and the indirect mannitol challenge have yielded conflicting results [3–29]. Several studies show that the two challenges have unexpectedly comparable sensitivity for asthma [7, 12, 13, 15] whereas other studies support the consensus that methacholine is more sensitive for a diagnosis of asthma [19, 22, 25, 26, 29]. A possible explanation is the observation from numerous studies that methacholine methods using a dosimeter with total lung capacity (TLC) inhalation (with a breath hold) demonstrate a marked loss of diagnostic sensitivity [30–32] due to deep inhalation bronchoprotection. This results in false negative challenges occurring in as many as 25% of overall methacholine tests and approaching 50% in asthmatics with mild AHR [33].

We hypothesized that deep inhalation methacholine methods with resulting bronchoprotection may be the explanation for conflicting sensitivity/specificity data. We have compared the diagnostic performance of the two challenges by examining studies where the two tests were performed in the same individuals (mostly) and where the methacholine inhalation method was clearly described.

## Methods

### Saskatoon studies

We began by identifying 46 unique individuals from four studies performed in our laboratory. We included the 20 subjects from the most recent study [29], 18 (of 20) additional subjects from a second study [26] and 8 (of 20) subjects from two allergen challenge studies [27, 28]. For analysis we selected the first methacholine challenge performed in the four studies, the only mannitol study by the standard method [3] from 2 studies [26, 29] and the pre-allergen mannitol challenge from the two allergen challenge studies [27, 28]. The methacholine challenges were done with the two minute tidal breathing method [34] in three studies [26–28] and by the tidal breathing vibrating mesh nebulizer volumetric method (0.5 mL methacholine nebulized to completion, 1.5 to 2.5 min tidal breathing) [35] in one [29]. A normal result is a provocation concentration causing a fall in forced expired volume in 1 s (FEV_1_) of 20% (PC_20_) of > 16 mg/mL for the former method [34] and non-cumulative provocation dose causing a 20% FEV_1_ fall (PD_20_) of > 400 μg for the latter [35]. For analysis, PC_20_ values were converted to PD_20_s based on the validated relationship that a PC_20_ of 16 mg/mL equates to a post evaporation non-cumulative PD_20_ of 400 μg [35–38]. A normal (negative) mannitol result is a cumulative PD_15_ > 635 mg [3]. Mannitol responsiveness was also assessed as the dose–response slope (DRS) so that a value was available for all individuals. Fractional exhaled nitric oxide (FeNO) [39] was available for all individuals. Data were analyzed with a computerized statistics programme, (Statistix 9 Analytical Software, Tallahassee, FL, USA). All data were log transformed. Log methacholine PD_20_ was compared to log mannitol DRS with linear regression and both log methacholine PD_20_ and log mannitol DRS were regressed with log FeNO.

### Other studies

Through a PubMed search, we identified 23 additional studies [3–25] that met the following criteria:

1. Mannitol testing was performed by the standardized protocol and results reported as the PD_15_ [3].
2. Methacholine challenges by various methods were done in the same subjects, with one exception where more subjects had methacholine tests than mannitol tests [25].
3. The methacholine inhalation method was described.
4. The definitions of “asthma” and “non-asthma” were outlined.

## Results

### Saskatoon studies

**Fig. 1:**
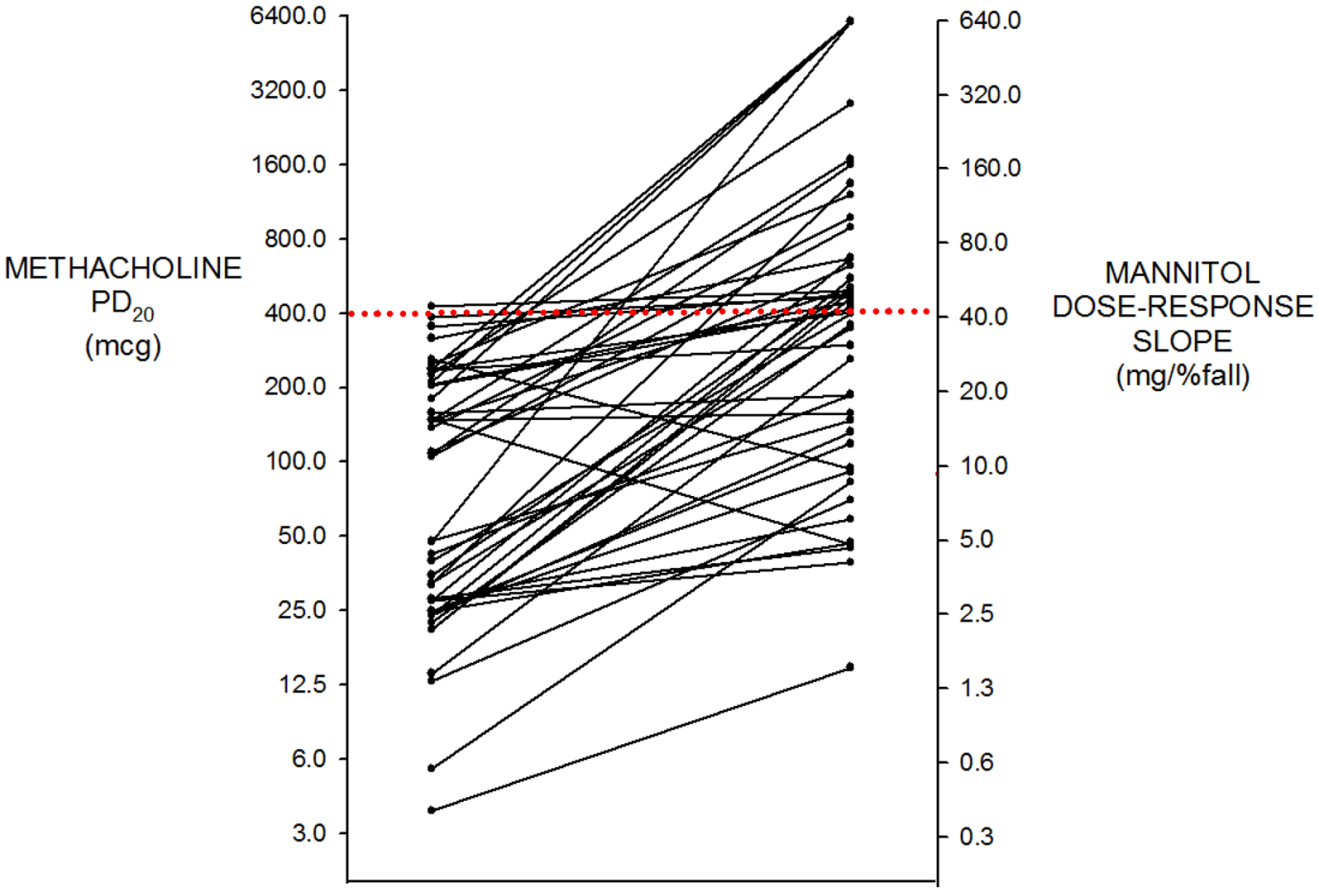
Individual data for methacholine PD_20_ in (μg) on the left and mannitol dose response slope (mg/% FEV_1_ fall) on the right. All values log transformed for analysis. The dotted red line, methacholine PD_20_ of 400 μg and mannitol DRS 42.3 (= mannitol PD_15_ of 635 mg), represents the cut points below which subjects are considered to have AHR to methacholine and mannitol respectively

**Fig. 2:**
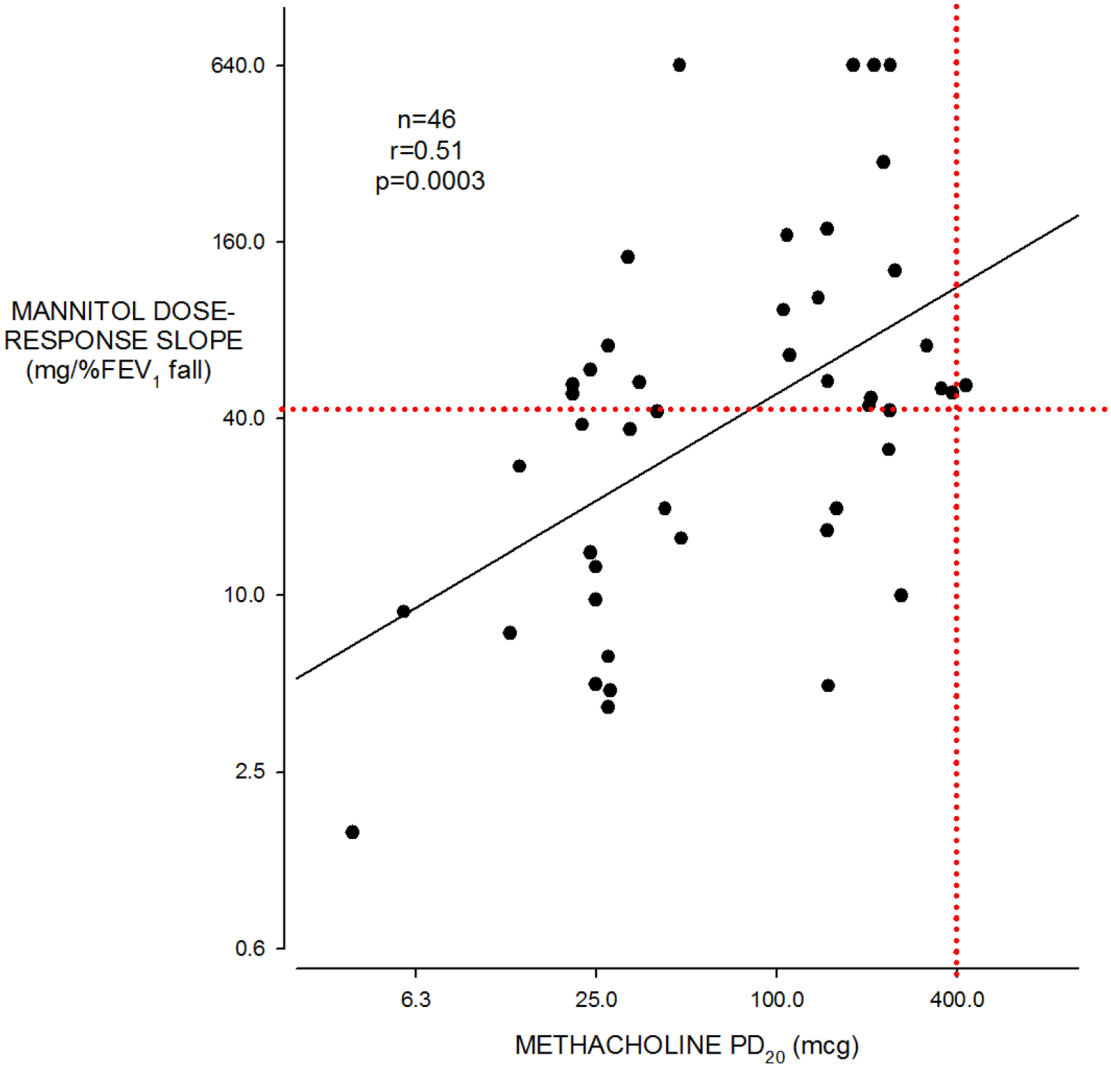
Mannitol DRS (mg/% FEV_1_ fall) on the vertical axis) and methacholine PD_20_ (μg) on the horizontal axis. The dotted red lines indicate the cut points below which the values indicate AHR to mannitol (42.3 mg/% FEV_1_ fall) or methacholine (400 μg) respectively. All values log transformed for analysis

**Fig. 3:**
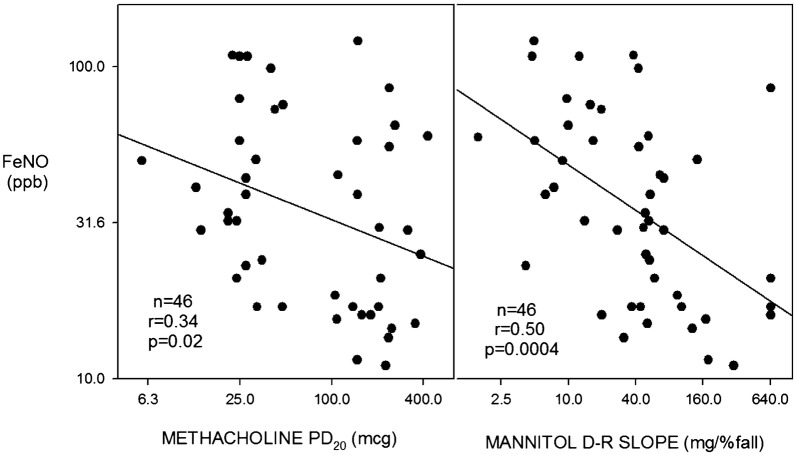
Correlation of FeNO on the vertical axis with methacholine PD_20_ (μg) on the horizontal axis left panel and mannitol DRS (mg/% FEV1 fall) on the horizontal axis right panel. All values log transformed

All 46 subjects had mild asthma and were not using inhaled corticosteroids (ICS). Age = 26.5 ± 8.5 (SD) years, height = 170 ± 9.6 cm, FEV_1_ = 3.45 ± 0.75 L and 91.5 ± 11.2% predicted. The methacholine PD_20_ was ≤ 400 μg in 45 of 46 (Fig. 1) and the geometric mean was 68.0 (95% CI 47.8–97.0) μg. The mannitol challenge was positive (PD_15_ ≤ 635 mg cumulative dose [3]) in 22 of 46. The 635 mg PD_15_ cut off equates to a DRS of 42.3 (mg/%fall) (Fig. 1). There was a moderate positive correlation between log methacholine PD_20_ and log mannitol DRS (r = 0.51, p = 0.0003, Fig. 2). Both log methacholine PD_20_ and log mannitol DRS correlated significantly and negatively with log FeNO (r = 0.34 and r = 0.50, respectively, Fig. 3): The correlation with FeNO was stronger for mannitol (p = 0.0004) than for methacholine (p = 0.02).

### Methacholine dosimeter TLC studies

Of 27 studies where methacholine and mannitol were compared [3–29] 13 used dosimeter TLC methods for methacholine inhalation [3–15]. These 13 studies are summarized in Table 1. The cut point for defining a positive methacholine test ranged from a cumulative PD_20_ of 7.8 to 10.2 μmol [3–6, 8–10, 14], or a non-cumulative PC_20_ of 8 [12] or 16 [7, 11, 13, 15] mg/mL (Table 2). Assuming nebulizer characteristics similar to the methods outlined by the ATS in 2000 [40], these would equate approximately to a non-cumulative post-evaporation PD_20_ between 200 and 400 μg. Four investigations studied known asthmatics [3, 6, 8, 15]; in one of these [3] asthma was defined by indirect AHR to hypertonic saline. Four studies involved subjects with “doctor diagnosed asthma” [5, 9, 12, 13], while three other studies defined asthma from a cohort with non-diagnostic symptoms, by a respiratory physician [7, 10] or panel [14] blinded to AHR data, and the final study defined asthma based on a positive AHR test (mannitol or methacholine) [11]. The non-asthmatic cohorts included subjects remaining in 5 studies after asthmatics had been defined [5, 7, 9, 10, 14], one study with normal controls [13], one study with a highly select group of asymptomatic (non-asthmatic) individuals with positive methacholine tests [4] and one study where non-asthma was define by negative AHR to both methacholine and mannitol [11].

**Table 1:** Mannitol compared to methacholine deep inhalation studies

	Refs		n	Asthma definition	n	Non-asthma definition
	Author	Ref				
1	Anderson et al.	[3]	25	Asthma with indirect AHR to hypertonic saline	0	
2	Pjorsgerg et al.	[4]	0		16	Asymptomatic positive MCT
3	Miedinger et al.	[5]	14	Asthma defined by board physician from 101 Swiss firefighters	87	Defined by board MD
4	Pjorsberg et al.	[6]	53	Asthmatics not using ICS	0	
5	Anderson et al.	[7]	240	240 of 375 with symptoms and unconfirmed asthma diagnosis made by AHR-blinded physician	135	Defined by AHR-blinded physician
6	Gade et al.	[8]	48	Asthmatics same day tests in random order 21 using ICS	0	
7	Miedinger et al.	[9]	42	Doctor diagnosed (MD-Dx) asthma 235 Swiss armed forces conscripts	193	Non-asthmatic conscripts
8	Sverrild et al.	[10]	51	From 238 randomly selected subjects Dx by physician blinded to AHR results	187	Blinded physician
9	Cancelliere et al.	[11]	11	From 28 with asthma-like Sx Dx defined by positive AHR	17	Defined by negative AHR
10	Manoharan et al.	[12]	123	MD-Dx asthma	0	
11	Kim et al.	[13]	50	MD-Dx asthma	54	Normal controls
12	Backer et al.	[14]	122	From 190 referred for possible asthma Dx by panel without AHR results	68	Defined by panel without AHR results
13	Park et al.	[15]	134	Asthmatic children 32 using ICS	0

**Table 2:** Mannitol compared to methacholine deep inhalation methods and results

	MCH method	Definition of positive MCH	Asthma		Asthma		Non Asthma		Non Asthma	
			MCH +ve	MCH total	MAN +ve	MAN total	MCH +ve	MCH total	MAN +ve	MAN total
1	DeVilbiss 40	PD20 ≤ 7.8 μmol^a^	25	25	25	25				
2	Nebicheck Dosimeter	PD20 ≤ 8.0 μmol^a^					16	16	1	16
3	Mefar dosimeter	PD20 ≤ 2 mg^a^–10.2 μmol	9	11	12	13	7	86	3	86
4	Nebicheck Dosimeter	PD20 ≤ 8.0 μmol^a^	43	53	43	53				
5	ATS Dosimeter	PC20 ≤ 16 mg/mL	122	240	132	240	34	135	36	135
6	Mefar dosimeter^b^	PD20 ≤ 2 mg^a^–10.2 μmol	22	48	22	48				
7	Spira dosimeter	PD20 ≤ 8.0 μmol^a^	18	42	17	42	15	193	14	193
8	Spira dosimeter	PD20 ≤ 8.0 μmol^a^	35	51	30	51	37	187	3	187
9	Spira dosimeter	PC20 ≤ 16 mg/mL	9	11	10	11	0	17	0	17
10	Mefar dosimeter	PC20 ≤ 8 mg/mL	74	123	76	123				
11	Chai dosimeter	PC20 ≤ 16 mg/mL	22	50	24	50	1	54	4	54
12	Jaeger dosimeter	PD20 ≤ 7.8 μmol^a^	79	122	46	122	32	68	11	68
13	Spira dosimeter	PC20 ≤ 16 mg/mL	90	134	100	134				
	Total %		548 60.2%	910	537 58.9%	912	142 18.8%	756	72 9.5%	756
	Exclude studies 1, 2 and 9 Total %		514 58.8%	874	502 57.3%	876	126 17.4%	723	71 9.8%	723

^a^PD_20_ Calculated cumulatively

^b^PD_20_ and PD_15_ calculated manually form response-dose ratio slope graphs

Results from the 12 asthma studies (Table 2) show similar sensitivity with positive methacholine tests in 60.2% (548 of 910) asthmatics and positive mannitol tests in 58.9% (537 of 912) asthmatics. When the two studies in which asthma was defined based on presence of AHR [3, 11] were excluded, the results were similar with 58.8% and 57.3% positive for methacholine and mannitol respectively (Table 2). In the 8 studies with non-asthma cohorts [4, 5, 7, 9–11, 13, 14], there were approximately twice as many positive methacholine tests (18.8% or 142 of 756) compared to mannitol tests (9.5% or 72 of 756) Table 2) Excluding the two studies in which AHR was either an inclusion [4] or an exclusion [11] criterion produced similar results, 17.4% and 9.8% positive for methacholine and mannitol respectively (Table 2).

### Methacholine tidal breathing studies

The 13 studies using tidal breathing methacholine methods [16, 18–29] compared to mannitol are summarized in Table 3. A fourteenth study that used histamine as the direct stimulus was also included [17]. Methacholine was inhaled by 2 min of tidal breathing from a jet nebulizer in 9 studies [16, 19, 21, 22, 24–28] or from a vibrating mesh nebulizer in one study [29]. The remaining four studies were defined as tidal breath dosimeter methods [17, 18, 20, 23]. The cut point definitions for a positive methacholine test (Table 4) included a cumulative PD_20_ of 1 to 2 mg (5.1–10.2 μmol) [17, 18, 20] or 8 μmol [23], a non-cumulative PC_20_ of 8 [23] or 16 [16, 19, 21, 24–28] mg/mL and a non-cumulative post-evaporation PD_20_ of 400 μg [29]. Once again, assuming nebulizer characteristics similar to the methods outlined by the ATS in 2000 [40] these would equate approximately to a non-cumulative post-evaporation PD_20_ between 200 and 400 μg. Known asthmatics were evaluated in 11 studies [16, 17, 20–22, 25–29] doctor diagnosed asthma in athletes in two studies [18, 23] and, from a group of symptomatic subjects, asthma diagnosed by a respiratory physician prior to AHR determination in one study [24] (Table 4). The 7 studies involving non-asthmatic cohorts included non-asthmatic controls in four [16, 17, 20, 25], the athletes remaining after doctor diagnosed asthma had been defined in two [18, 23], and the symptomatic individuals remaining after asthma was diagnosed [24] (Table 4).

**Table 3:** Mannitol compared to methacholine tidal breathing studies

	Reference		n	Asthma definition	n	Non asthma definition
	Author	Ref #				
1	Subbarao et al.	[16]	25	Asthmatic children with positive methacholine test	10	Non asthmatic methacholine negative
2	Koskelka et al.	[17]	37	Mild corticosteroid naïve asthmatics NB: Histamine	10	Non asthmatic controls
3	Sue-Chu et al.	[18]	10	MD-Dx asthma from 58 cross country skiers	48	Non asthmatic cross country skiers
4	Andregnette et al.	[20]	30	Current asthmatic children	0	
5	Aronsson et al.	[19]	34	Asthmatics	18	Non asthmatic controls
6	Lemiere et al.	[21]	30	Occupational asthmatics	0	
7	Andregnette et al.	[22]	23	Asthmatic children with EIB symptoms	0	
8	Toennesen et al.	[23]	18	MD-Dx asthma from 57 elite athletes	39	Non asthmatic Elite athletes
9	Porpodis et al.	[24]	67	From 88 subjects with asthma-like symptoms	21	Symptoms but no asthma
10	Gutierrez et al.	[25]	156	Asthmatic children	38	Non asthmatic controls
11	Cockcroft et al.	[26–28]	26	Mild asthma no ICS	0	
12	Blais et al.	[29]	20	Mild asthma no ICS	0

**Table 4:** Mannitol compared to methacholine tidal breathing methods and results

	MCH Method	Definition of positive MCH	Asthma		Asthma		Non asthma		Non asthma	
			MCH +ve	MCH total	MAN +ve	MAN total	MCH +ve	MCH total	MAN +ve	MAN total
1	2 min TB (ref Cockcroft et al [34])	PC20 ≤ 16 mg/mL	25	25	21	25	0	10	0	10
2	Spira tidal dosimeter	PD20 ≤ 1 mg^a^ (Hist.)	30	37	19	37				
3	Spira tidal dosimeter	PD20 ≤ 1814 μg^a^	4	10	2	10	19	48	1	48
4	TB (ref Cockcroft et al [34])	PC20 ≤ 16 mg/mL	29	30	13	30				
5	Jaeger tidal dosimeter	PD20 ≤ 2 mg^a^	27	34	13	34	3	18	0	18
6	2 min TB (ref Cockcroft et al [34])	PC20 ≤ 16 mg/mL	22	30	9	30				
7	TB (ref Cockcroft et al [34])	PC20 ≤ 8 mg/mL	18	23	10	23				
8	Spira tidal dosimeter	PD20 ≤ 8 μmol^a^	15	16	9	18	1	39	3	39
9	TB (ref Cockcroft et al [34])	PC20 ≤ 16 mg/mL	42	67	43	67	3	21	0	21
10	2 min TB (ref Cockcroft et al [34])	PC20 ≤ 16 mg/mL	131	141	7	77	1	30	0	21
11	2 min TB (3 studies) [34]	PC20 ≤ 16 mg/mL	25	26	11	26				
12	Solo TB (1.5–2.5 min) [35]	PD20 ≤ 400 μg	20	20	11	20				
	Total %		343 83.1%	413	146 41.5%	351	27 16.2%	166	4 2.5%	157

^a^PD_20_ calculated cumulatively

Results are summarized in Table 4. Methacholine tests were more than twice as likely to be positive in asthmatics (i.e. methacholine more sensitive) than was mannitol. The positive rate was 83.1% (343 of 413) for methacholine and 41.5% (146 of 351) for mannitol. In the non-asthmatics methacholine was more likely to be positive at 16.2% (27 of 166) than was mannitol at 2.5% (4 of 157).

When both methacholine TLC and methacholine TB studies were combined, the overall rate of a positive mannitol challenge in non-asthmatics was 8.3% or 76 of 913.

## Discussion

These data provide strong support for the hypothesis that tidal breathing direct methacholine challenge methods yield results that are substantially more sensitive for asthma than does the indirect mannitol challenge. By contrast, when methacholine is inhaled by TLC methods, the diagnostic sensitivity falls to a level similar to that seen with mannitol.

Many investigators have found that AHR correlates with airway inflammation, primarily with eosinophils, as assessed by broncho-alveolar lavage (BAL), induced sputum cell counts or indirectly by FeNO or blood eosinophils [41–47]. Initial studies addressed methacholine (direct) AHR and BAL eosinophils and metachromatic cells (basophils and mast cells) [41, 42]. Subsequent studies addressed, in addition, indirect challenges, AMP [43, 44], bradykinin [45] and mannitol [26, 29, 46, 47]. While these investigations show a fair to good correlation between methacholine AHR and primarily eosinophilic inflammation, the indirect AHR tests correlate substantially better with inflammation [43–46]. The results from our combined investigations [26–29], using FeNO as an indirect measure of eosinophilic airway inflammation, are in keeping with this as shown in Fig. 3. Relatively few studies have addressed the potentially more important [48] metachromatic cells (mast cells and/or basophils) [41, 42, 47]. There is a hint from these studies that airway metachromatic cell inflammation may correlate better with AHR than does eosinophilic airway inflammation.

AHR improves with anti-inflammatory therapeutic strategies including allergen avoidance environmental control [49, 50] and ICS [51–53]. In keeping with the above observations, indirect AHR (AMP [49–52]) shows greater improvement with these treatments than does direct methacholine AHR. Mannitol responsiveness improves greatly after ICS treatment [53] and can provide a useful predictive marker of a pending asthma exacerbation during ICS tapering [54]. Although direct AHR has been proposed to monitor and guide asthma treatment [55], indirect AHR may provide a particularly valuable tool as a guide to monitoring asthma control [56]. In fact, non-responsiveness to indirect challenge (e.g. AMP, mannitol) may be a goal for adequate asthma control with ICS [56]. This, of course, is consistent with a positive indirect AHR challenge (including mannitol) being insensitive for the diagnosis of well controlled asthma.

Deep inhalations to TLC produce potent bronchodilation and bronchoprotection, the latter greater than the former, in normal individuals but initially stated to not occur in asthmatics [57]. It had become apparent that this marked bronchoprotective effect extends to mild asthmatics [30–33] and, in all likelihood may well extend to well controlled asthmatics. Although not seen in all studies [58], eosinophilic airway inflammation impairs the bronchoprotective effect of deep inhalation [26, 59, 60]. Anti-inflammatory strategies, both allergen avoidance [61] and oral/inhaled corticosteroid [62], can restore or improve the deep inhalation bronchoprotection in asthmatics. In one study, lack of bronchoprotection (methacholine) and elevated levels FeNO as an indirect measure of airway inflammation were associated with indirect AHR to mannitol [26].

Collectively, these data suggest that airway inflammation (eosinophilic particularly), indirect AHR and loss of deep inhalation bronchoprotection will occur together in asthmatics. Conversely, deep inhalation bronchoprotection and low levels of airway inflammation will be associated with little if any indirect AHR [26]. Avoidance of TLC inhalations during methacholine inhalation will therefore result in many more positive direct challenge tests in mild (and possibly well controlled) asthmatics with no indirect AHR and minimal airway inflammation. This is confirmed by our current review.

Deep inhalation bronchoprotection during methacholine challenges is an important and underappreciated phenomenon [33]. This has been shown by three studies from our laboratory [30–32] and supported by studies from other laboratories [63, 64]. This was first suggested in a study of 40 individuals [30] comparing the two methacholine methods outlined in the ATS document [40]. Follow up investigations demonstrated that asthmatics with negative TLC dosimeter methacholine tests had positive challenges when the identical dosimeter dose was administered with sub-maximal inhalations (approximately half TLC) [31] and that many asthmatics with positive tidal breathing methacholine challenges were negative when five TLC breaths were incorporated at equal intervals throughout the 2 min of tidal breathing [32]. These latter two studies provide convincing evidence of the bronchoprotective effect of deep TLC inhalations in many individuals with mild asthma. Our summary data from 55 asthmatic individuals with positive tidal breathing methacholine tests revealed that 13 (24%) had negative five TLC breath dosimeter methacholine tests [33]. This represents 50% of asthmatics with a tidal breathing PC_20_ between 2 and 16 mg/mL (post evaporation non-cumulative PD_20_ between 50 and 400 μg). This is exactly the range where a positive diagnostic methacholine challenge, done in individuals with symptoms suggestive of asthma and normal spirometry, is likely to fall. In this population, the TLC dosimeter methacholine method could, therefore, produce a false negative rate approaching 50% for individuals with asthma and mild AHR. For these reasons the recent methacholine guidelines have strongly suggested that methacholine challenges be performed with tidal breathing methods with a non-TLC dosimeter method as a second option [36]. By contrast, as anticipated by the above data, our recent study documented that removal of TLC inhalations from the mannitol challenge did not affect the result [29].

It is difficult to accurately comment on sensitivity and specificity of the different tests from the available references. A reasonable estimate of diagnostic sensitivity can be made by assessing the rate of positivity in subjects determined to have asthma. Based on this approach the tidal breathing methacholine test is about twice as sensitive for “asthma” as the mannitol test (83.1% and 41.5% respectively) in the studies assessed, whereas the sensitivities of TLC methacholine and mannitol tests were similar, at approximately 60% for both in the studies included. These data suggest that the loss of diagnostic sensitivity of the methacholine test when using a TLC dosimeter method is significant enough to make the sensitivity equivalent to an indirect challenge. It is even more difficult to comment accurately on specificity without a larger cohort of normal non-asthmatic individuals. The observation that there were fewer positive mannitol tests (about half) compared to methacholine tests in non-asthmatics is consistent with the consensus that indirect challenges, including mannitol, are more specific for asthma [2, 65]. The difficulties are further compounded both by the lack of an independent gold standard for the diagnosis of asthma and by the requirement for the symptoms under investigation to be clinically current, i.e. within the past few days [65, 66].

We suspect that these results would translate to indirect challenges other than mannitol; these include AMP, propranolol, hypertonic saline, EVH and exercise (EIB). It is likely that all these indirect challenges would show minimal if any deep inhalation bronchoprotection. EVH and EIB are particularly important. It would, however, be difficult to design a study with and especially without deep inhalations for these two, especially for EVH.

Indirect challenges require a substantially larger dose of stimulus than direct challenges, up to or greater than three orders of magnitude mg for mg or mmol for mmol [65]. For example, the top doses for mannitol and methacholine are 635 (cumulative) and 0.4 mg (non-cumulative) respectively. It is possible that mannitol might be more sensitive than many other indirect stimuli because the challenge is less likely to be “dose limited” [65]. There are physiologic limits on the “dose” of stimulus that can be achieved with exercise or EVH, and, because of the large doses needed, a solubility limit on the doses that can be achieved with AMP or propranolol [65]. Mannitol, by contrast, is a dry powder inhalation and the dose is not limited by solubility. There is only one mannitol inhalation method [3]. However, the large number of different methacholine methods represents a difficulty when attempting to compare data. A conservative estimate is that there were at least 6 different TLC dosimeter methods and 4 different TB methods in the studies evaluated. The best case estimate is that these methods equated to a post-evaporation methacholine PD_20_ range of only twofold (200–400 μg), however that is speculation without knowledge of the operating characteristics of the different nebulizers used.

## Conclusion

The discordance between methacholine and mannitol comparisons can be explained by the method of methacholine inhalation. Tidal breathing methacholine tests are substantially more sensitive than mannitol tests for a diagnosis of asthma and equally more sensitive than TLC dosimeter methacholine methods. In order to preserve a high diagnostic sensitivity, methacholine challenges should be performed by tidal breathing [33, 36, 65], thus providing data that are complementary to the more specific mannitol challenge.

## Data Availability

All data are available from the corresponding author on reasonable request don.cockcroft@usask.ca.

## Abbreviations

FEV_1_: Forced expired volume in 1 s
PC_20_: Provocation concentration causing a 20% FEV_1_ fall
PD_20_: Provocation dose causing a 20% FEV_1_ fall
MCH: Methacholine
MAN: Mannitol
AHR: Airway hyperresponsiveness
DRS: Dose response slope
TLC: Total lung capacity
TB: Tidal breathing
EVH: Eucapnic voluntary hyperpnea
AMP: Adenosine monophosphate
FeNO: Fractional exhaled nitric oxide
ICS: Inhaled corticosteroid
MD-Dx: Doctor diagnosed
SD: Standard deviation
CI: Confidence interval
BAL: Broncho-alveolar lavage
